# Five-minute Apgar score and risk of neonatal mortality, severe neurological morbidity and severe non-neurological morbidity in term infants – an Australian population-based cohort study

**DOI:** 10.1016/j.lanwpc.2024.101011

**Published:** 2024-01-13

**Authors:** Jesrine Hong, Kylie Crawford, Kate Jarrett, Tegan Triggs, Sailesh Kumar

**Affiliations:** a Mater Research Institute, University of Queensland, Level 3, Aubigny Place, Raymond Terrace, South Brisbane, Queensland, 4101, Australia; b School of Medicine, The University of Queensland, Herston, Queensland, 4006, Australia; c Department of Obstetrics and Gynecology, Faculty of Medicine, Universiti Malaya, Kuala Lumpur, 50603, Malaysia; d NHMRC Centre for Research Excellence in Stillbirth, Mater Research Institute, University of Queensland, Brisbane, Queensland, Australia

**Keywords:** Apgar score, Neonatal mortality, Neonatal morbidity, Neurological, Term infant

## Abstract

**Background:**

The aim of this study was to ascertain risks of neonatal mortality, severe neurological morbidity and severe non-neurological morbidity related to the 5-min Apgar score in early term (37^+0^–38^+6^ weeks), full term (39^+0^–40^+6^ weeks), late term (41^+0^–41^+6^ weeks), and post term (≥42^+0^ weeks) infants.

**Methods:**

This was a retrospective cohort study of 941,221 term singleton births between 2000 and 2018 in Queensland, Australia. Apgar scores at 5-min were categorized into five groups: Apgar 0 or 1, 2 or 3, 4–6, 7 or 8 and 9 or 10. Gestational age was stratified into 4 groups: Early term, full term, late term and post term. Three specific neonatal study outcomes were considered: 1) Neonatal mortality 2) Severe neurological morbidity and 3) Severe non-neurological morbidity. Poisson multivariable regression models were used to determine relative risk ratios for the effect of gestational age and Apgar scores on these severe neonatal outcomes. We hypothesized that a low Apgar score of <4 was significantly associated with increased risks of neonatal mortality, severe neurological morbidity and severe non-neurological morbidity.

**Findings:**

Of the study cohort, 0.04% (345/941,221) were neonatal deaths, 0.70% (6627/941,221) were infants with severe neurological morbidity and 4.3% (40,693/941,221) had severe non-neurological morbidity. Infants with Apgar score <4 were more likely to birth at late term and post term gestations and have birthweights <3rd and <10th percentiles. The adjusted relative risk ratios (aRRR) for neonatal mortality and severe neurological morbidity were highest in the Apgar 0 or 1 cohort. For infants in the Apgar 0 or 1 group, neonatal mortality increased incrementally with advancing term gestation: early term (aRRR 860.16, 95% CI 560.96, 1318.94, p < 0.001); full term (aRRR 1835.77, 95% CI 1279.48, 2633.91, p < 0.001); late term (aRRR 1693.61, 95% CI 859.65, 3336.6, p < 0.001) and post term (aRRR 2231.59, 95% CI 272.23, 18293.07, p < 0.001) whilst severe neurological morbidity decreased as gestation progressed: early term (aRRR 158.48, 95% CI 118.74, 211.51, p < 0.001); full term (aRRR 112.99, 95% CI 90.56, 140.98, p < 0.001); late term (aRRR 87.94, 95% CI 67.09, 115.27, p < 0.001) and post term (aRRR 52.07, 95% CI 15.17, 178.70, p < 0.001). Severe non-neurological morbidity was greatest in the full term, Apgar 2–3 cohort (aRRR 7.36, 95% CI 6.2, 8.74, p < 0.001).

**Interpretation:**

A 5-min Apgar score of <4 was prognostic of neonatal mortality, severe neurological morbidity, and severe non-neurological morbidity in infants born >37 weeks’ gestation with the risk greatest in the early term cohort.

**Funding:**

10.13039/501100000925National Health and Medical Research Council and 10.13039/100015471Mater Foundation.

**Research in context**

**Evidence before this study**

In term infants, the 5-min Apgar score, particularly if <4 is associated with an increased risk of neonatal mortality, infant mortality, short and long-term neurological impairment. In preterm infants however, it has limited prognostic utility. The risk of neonatal and infant death decreases with increasing gestational age, however, infants born at early term gestation have a greater risk of severe neonatal morbidity and poorer long-term neurodevelopmental outcomes compared to full-term infants. In Australia, rates of low 5-min Apgar (<4) score are almost 10 times greater in Indigenous newborns compared to the general population.

We searched PubMed for articles evaluating risk of severe neonatal morbidity in relation to Apgar score and gestational age among term infants up to June 1, 2023, using the search terms “Apgar score”, “term”, “risk”, “severe neonatal morbidity”, and “gestational age”. We found no observational study assessing the relationship between the Apgar score at 5-min, severe neurological morbidity and severe non-neurological morbidity in term infants stratified by gestational age group.

**Added value of this study**

In this large Australian cohort study, we provide more granularity of information by stratifying Apgar scores and term gestational age categories to show the combined effect of both on severe adverse neonatal outcomes. Poisson multivariable regression models were used to determine relative risk ratios for the effect of gestational age and Apgar scores on these outcomes. We found that low 5-min Apgar scores were associated with increased risk of neonatal death, severe neurological morbidity as well as severe non-neurologic neonatal morbidity across all term gestational age categories. The adjusted relative risks of neonatal mortality were highest in the post-term gestational age group whilst for severe neurological morbidity they were greatest in the early term cohort. As for severe non-neurological morbidity, the risks were broadly similar for all Apgar score groups regardless of gestational age, with the highest risk present when the Apgar score was <4.

**Implications of all the available evidence**

Our results extend the findings of previous studies by showing a dose–response associations between Apgar score and risks of severe neurological and severe non-neurological neonatal morbidity across term gestational age categories.

## Introduction

The Apgar score reflects a standardized assessment of an infant's color, heart rate, reflexes, muscle tone and respiratory effort after birth.^1^ It is a globally used, easy and convenient method for reporting the status of the newborn infant and its response to resuscitation. However, it is only representative of the infant's physiologic condition at a single time point and may be influenced by various factors including gestational age, inter-observer variability or the presence of structural, chromosomal or genetic abnormalities.^1^^,^^2^ Intrapartum factors associated with moderate (4–6) or low (<4) Apgar scores include maternal sedation or anesthesia, vaginal breech birth, birth trauma, meconium staining of liquor, mode and urgency of operative birth.^3^^,^^4^ A low score may also be indicative of substandard care during labor.^5^

Although both 1 and 5-min scores are predictors for neonatal morbidity, the 5-min score is accepted as a more useful predictor of outcome irrespective of birth weight.^1^ An Apgar score of <7 at 5-min after birth is associated with an increased risk of neonatal mortality,^6^^,^^7^ infant mortality,^6^ cardiovascular diseases in childhood,^8^ short and long-term neurological morbidities^9^ in term infants. A low score of <4 however, is a sign of significant compromise and one of the first indications of encephalopathy in term and late-preterm infants and potentially predictive of longer term neurodevelopmental impairment.^2^^,^^10^, ^11^, ^12^ However, in more preterm infants recent data suggests that the 5-min Apgar score may not be as prognostic for long-term motor and cognitive outcomes as previously believed.^13^

In term infants, the risk of neonatal and infant death decreases with increasing gestational age, however, infants born at early term gestation (37^+0^–38^+6^ weeks) have a greater risk of severe neonatal morbidity,^14^ increased rates of hospital admission during childhood^14^^,^^15^ and poorer long-term neurodevelopmental outcomes^16^ compared to full-term infants.^17^ There is however limited information regarding the association between the 5-min Apgar score and severe neonatal outcomes stratified by gestational age in term infants. Thus, the specific aims of this study were to investigate the relationship between the Apgar score at 5-min, neonatal mortality, severe neurological morbidity and severe non-neurological morbidity in early term, term, late term and post term infants using a large Australian perinatal dataset.

## Methods

### Study design and population

This was a retrospective cohort study of singleton births between 2000 and 2018 in Queensland, Australia. The Queensland Perinatal Data Collection^18^ was provided by Statistical Output and Reporting Unit, Statistical Services Branch of Queensland's Department of Health. This dataset contains deidentified maternal and perinatal data of all births in Queensland. Institutional ethical approval was granted by the Metro North Hospital and Health Service Human Research Ethics Committee (Reference number: LNR/219/QRBC/53154). Only singleton, term live births (>37^+0^ weeks' gestation) with documented Apgar scores at 5-min were included. Infants with major structural abnormalities, known chromosomal or genetic disorders were excluded.

### Exposure

Five-minute Apgar scores were categorized into five ordinal groups similar to that previously described by Cnattingius et al.^19^: Apgar 0 or 1, 2 or 3, 4–6, 7 or 8 and 9 or 10. The highest of these ordinal groups (Apgar score 9 or 10) was used as the referent category for evaluating the incremental dose response relationships between 5-min Apgar scores and outcomes. A low Apgar score was defined as a score of 0–3, moderately abnormal as a score of 4–6, whilst a score of 7–10 was regarded as reassuring.^20^

### Outcomes

The three study outcomes were neonatal mortality, severe neurological morbidity and severe non-neurological morbidity diagnosed within 28-days of birth. To avoid interpretative difficulties arising from a pregnancy having multiple adverse perinatal outcomes, all outcomes were analyzed as binary variables and were considered as mutually exclusive according to the following ranking: 1) neonatal mortality 2) severe neurological morbidity and 3) severe non-neurological morbidity. We did not examine the exact time interval between birth and death because this granularity of data was not available. Severe neurological morbidity was defined as any infant recorded in the dataset as experiencing birth asphyxia, hypoxic ischemic encephalopathy, neonatal seizures or intraventricular hemorrhage. Severe non-neurological neonatal morbidity was defined as a composite of neonatal intensive care unit (NICU) admission for >24 h, neonatal sepsis (bacterial, viral and fungal) or necrotizing enterocolitis. Neonatal mortality and components of the severe neurological and non-neurological composite outcomes were obtained using International Classification of Diseases 10th Revision (ICD-10) codes as was recorded in the perinatal dataset. ICD-10 codes relevant to maternal health characteristics, intra-partum events and neonatal outcomes are detailed in Appendix Table S1. Our choice of components for the composite outcomes was guided by a recent publication^21^ which defined a core perinatal outcome set identified as important and meaningful by key international stakeholders including parents, midwives, nurses and allied health professionals, obstetricians, neonatologists, pediatricians, academics, and researchers. These outcomes are applicable to any research involving infants in a high-income setting and are intended to apply regardless of gestational age at birth, birth weight (BW), illness severity, specific infant population, clinical setting, or condition.

### Maternal demographic and intra-partum characteristics

We considered various maternal demographic and intra-partum characteristics (Table 1) that could potentially confound or modify the effect of 5-min Apgar score on the study outcomes. Socioeconomic status was defined according to the socioeconomic index for areas (SEIFA) score generated by the Australian Bureau of Statistics.^22^ This score ranks geographical regions according to socioeconomic advantage based on information from the five-yearly census with scores in the lowest quintile reflecting relative socioeconomic disadvantage. Rates of smoking, drug and alcohol use were established using a combination of self-reported variables and ICD-10 codes. BW centiles were defined according to Australian population charts.^23^ Small for gestational age (SGA) was defined as an infant with a BW <10th centile for gestation.^23^

**Table 1:** Maternal demographic characteristics and labor outcomes stratified by Apgar score.

Apgar score	Total	0 or 1	2 or 3	4 to 6	7 or 8	9 or 10
	N = 941,221	N = 388	N = 959	N = 8382	N = 43,587	N = 887,905
**Maternal health, demographic and obstetric history**						
Maternal age (years)	29 (25, 33)	29 (25, 34)	28 (24, 33)	29 (24, 33)	29 (24, 33)	29 (25, 33)
Nulliparous	378,746 (40.2%)	192 (49.5%)	509 (53.1%)	4580 (54.6%)	22,629 (51.9%)	350,836 (39.5%)
Born in Australia	740,505 (78.7%)	294 (75.8%)	761 (79.4%)	6611 (78.9%)	35,134 (80.6%)	697,705 (78.6%)
Indigenous	60,231 (6.4%)	28 (7.2%)	77 (8.0%)	682 (8.1%)	3028 (6.9%)	56,416 (6.4%)
Previous <20 weeks miscarriage	312,231 (33.2%)	147 (37.9%)	300 (31.3%)	2879 (34.3%)	14,443 (33.1%)	294,462 (33.2%)
Previous stillbirth	12,311 (1.3%)	6 (1.5%)	11 (1.1%)	95 (1.1%)	514 (1.2%)	11,685 (1.3%)
Previous caesarean	152,324 (16.2%)	48 (12.4%)	113 (11.8%)	1015 (12.1%)	5680 (13.0%)	145,468 (16.4%)
APH >20 weeks	19,956 (2.1%)	39 (10.1%)	55 (5.7%)	345 (4.1%)	1403 (3.2%)	18,114 (2.0%)
Smoking in pregnancy	110,349 (11.7%)	59 (15.2%)	144 (15.0%)	1226 (14.6%)	5628 (12.9%)	103,292 (11.6%)
Illicit drug use in pregnancy	5746 (0.6%)	5 (1.3%)	13 (1.4%)	123 (1.5%)	425 (1.0%)	5180 (0.6%)
Alcohol use	1664 (0.2%)	1 (0.3%)	4 (0.4%)	27 (0.3%)	99 (0.2%)	1533 (0.2%)
BMI ≥35 kg/m^2^	47,214 (8.0%)	31 (13.5%)	58 (8.8%)	653 (10.9%)	2831 (10.1%)	43,641 (7.9%)
Assisted conception	35,080 (3.7%)	9 (2.3%)	31 (3.2%)	295 (3.5%)	1600 (3.7%)	33,145 (3.7%)
Lowest SES quintile	190,593 (20.4%)	90 (23.5%)	244 (25.6%)	1982 (23.9%)	9406 (21.8%)	178,871 (20.3%)
Diabetes mellitus^a^	69,371 (7.4%)	31 (8.0%)	82 (8.6%)	814 (9.7%)	4009 (9.2%)	64,435 (7.3%)
Pre-eclampsia	51,049 (5.4%)	43 (11.1%)	86 (9.0%)	782 (9.3%)	3654 (8.4%)	46,484 (5.2%)
**Intrapartum and birth outcomes**						
Labor onset						
Spontaneous	521,752 (55.4%)	234 (60.3%)	513 (53.5%)	4563 (54.4%)	23,253 (53.3%)	493,189 (55.5%)
Induced	239,966 (25.5%)	106 (27.3%)	319 (33.3%)	2778 (33.1%)	13,952 (32.0%)	222,811 (25.1%)
Failed IOL	7026 (0.7%)	4 (1.0%)	6 (0.6%)	69 (0.8%)	383 (0.9%)	6564 (0.7%)
Total duration of labor (mins)	307 (183, 490)	370 (204, 563)	355 (216, 551)	396 (231, 600)	382 (225, 595)	304 (181, 483)
Second stage of labor ≥3 h	15,497 (2.4%)	16 (6.7%)	26 (4.3%)	242 (4.4%)	1337 (4.5%)	13,876 (2.3%)
Method of birth						
Spontaneous vaginal	561,732 (59.7%)	186 (47.9%)	499 (52.0%)	4093 (48.8%)	21,404 (49.1%)	535,550 (60.3%)
Instrumental vaginal	90,092 (9.6%)	52 (13.4%)	102 (10.6%)	1419 (16.9%)	8680 (19.9%)	79,839 (9.0%)
Forceps	22,525 (2.4%)	17 (4.4%)	24 (2.5%)	331 (3.9%)	2080 (4.8%)	20,073 (2.3%)
Vacuum	67,567 (7.2%)	35 (9.0%)	78 (8.1%)	1088 (13.0%)	6600 (15.1%)	59,766 (6.7%)
Emergency caesarean	117,882 (12.5%)	119 (30.7%)	263 (27.4%)	2038 (24.3%)	7826 (18.0%)	107,636 (12.1%)
Elective caesarean	171,513 (18.2%)	31 (8.0%)	95 (9.9%)	832 (9.9%)	5677 (13.0%)	164,878 (18.6%)
Analgesia during birth						
No analgesia	130,298 (13.8%)	58 (14.9%)	92 (9.6%)	687 (8.2%)	4014 (9.2%)	125,447 (14.1%)
Systemic opioids	71,535 (7.6%)	36 (9.3%)	139 (14.5%)	1157 (13.8%)	4549 (10.4%)	65,654 (7.4%)
Epidural/Spinal	435,833 (46.3%)	150 (38.7%)	340 (35.5%)	3731 (44.5%)	21,497 (49.3%)	410,115 (46.2%)
General anesthetic	18,314 (1.9%)	67 (17.3%)	179 (18.7%)	1107 (13.2%)	2911 (6.7%)	14,050 (1.6%)
NRFS	189,398 (20.1%)	187 (48.2%)	442 (46.1%)	3781 (45.1%)	17,068 (39.2%)	167,920 (18.9%)
Cord prolapse	1256 (0.1%)	13 (3.4%)	18 (1.9%)	75 (0.9%)	186 (0.4%)	964 (0.1%)
Intrapartum hemorrhage	1898 (0.2%)	3 (0.8%)	12 (1.3%)	44 (0.5%)	169 (0.4%)	1670 (0.2%)
Uterine rupture	262 (0.0%)	5 (1.3%)	9 (0.9%)	21 (0.3%)	40 (0.1%)	187 (0.0%)
Failed instrumental delivery	5796 (0.6%)	10 (2.6%)	11 (1.1%)	173 (2.1%)	707 (1.6%)	4895 (0.6%)
Cesarean for NRFS	36,349 (12.6%)	74 (49.3%)	166 (46.4%)	1105 (38.5%)	3577 (26.5%)	31,427 (11.5%)
Instrumental delivery for NRFS	36,379 (40.4%)	26 (50.0%)	55 (53.9%)	740 (52.1%)	4286 (49.4%)	31,272 (39.2%)
Cesarean for failure to progress	48,752 (16.8%)	25 (16.7%)	60 (16.8%)	562 (19.6%)	2526 (18.7%)	45,579 (16.7%)
Instrumental delivery for failure to progress	37,492 (41.6%)	16 (30.8%)	34 (33.3%)	469 (33.1%)	3042 (35.0%)	33,931 (42.5%)

Data are presented as median (IQR) for continuous measures, and (N) column % for categorical measures. APH, Antepartum Hemorrhage; BMI, Body Mass Index; IOL, Induction of Labor; NRFS, Non-Reassuring Fetal Status; SES, Socioeconomic Status.

a Diabetes includes: Gestational Diabetes, Type 1 and Type 2 Diabetes.

Demographic variables analyzed as binary variables included: Indigenous status (yes vs. no), parity (nulliparous vs. parity >1), high body mass index (BMI) (≥35 kg/m^2^ vs. <35 kg/m^2^), low socioeconomic status (lowest quintile of the SEIFA score vs. quintiles 2–5), smoking (yes vs. no), drug use (yes vs. no), diabetes (yes vs. no), preeclampsia (yes vs. no), antepartum hemorrhage (yes vs. no), intra-partum non-reassuring fetal status (yes vs. no) and small for gestational age (<10th centile vs. ≥10th centile). Maternal age was considered as a continuous variable as well as categorized into 3 groups (<25 years, 25–34 years and ≥35 years). Induction of labor was considered as a categorical variable with 3 groups (spontaneous, induced and no labor). Gestational age at birth was categorized into four groups: Early term (37^+0^–38^+6^ weeks), full term (39^+0^–40^+6^ weeks), late term (41^+0^–41^+6^ weeks) and post term (≥42^+0^ weeks). Method of birth was categorized into: spontaneous vaginal delivery, instrumental vaginal delivery, emergency Cesarean section and elective Cesarean section.

### Statistical analysis

Data were analyzed using Stata 18® (Statacorp, College Station, TX, USA). The distribution of continuous variables was assessed using histograms. Maternal demographic variables and intra-partum outcomes that could potentially confound or modify the effect of Apgar scores on neonatal outcomes are presented as median and interquartile ranges or numbers and column percentages.

### Unadjusted incidence rate differences for study outcomes

The incidence rates (per 100 births) for neonatal death, severe neurological morbidity and severe non-neurological morbidity were determined for each of the five Apgar score categories. The rate differences and 95% confidence intervals (95% CI) for each Apgar score category and the referent cohort (Apgar score 9–10) were calculated according to gestational age category at birth.

### Missing data

Missing values of BMI (351,614/941,221) were imputed using logistic regression, with gestational age, neonatal outcome, 5-min Apgar score, nulliparity, method of birth and pre-eclampsia as covariates. Imputation of missing data was deemed appropriate after cross-tabulations revealed similar proportions of the three study outcomes and Apgar score categories.

### Confounders

The same confounders were selected for all three outcomes, as all share common causal risk factors and are essentially gradations in severity. Potential maternal demographic and intra-partum confounders considered for inclusion in Poisson multivariable models were selected based on clinical relevance^24^^,^^25^ and if there was a change in coefficient in at least one outcome by a minimum of 10%.^26^ When covariates were reduced in stratified models to ensure sufficient events per variable for all outcomes,^25^ alternative confounding variables were further investigated in Supplementary analyses.

### Poisson multivariable analyses

Poisson multivariable regression models were used to determine relative risk ratios (RRR) and 95% CI for the effect Apgar scores on the study outcomes, with the gestational age at birth (in weeks) as the time variable. We used Poisson models rather than binary regression analysis because gestational age at birth can influence both the Apgar score and the risk of adverse neonatal outcomes.^13^ Furthermore, odds ratios from binary regression may not accurately represent the relative risk if the outcome is not rare.^27^ Multivariable models were built to determine: 1) The association between Apgar score categories and neonatal death, severe neurological morbidity and severe non-neurological morbidity and 2) The association between Apgar score categories and adverse neonatal outcomes within strata of term gestational age categories. Robust standard errors were used to account for clustering at the maternal level because new pregnancies in the same woman are assigned new identification numbers in the Queensland Perinatal Dataset. Goodness-of-fit was tested using post-estimation Deviance and Pearson chi-squared statistics.^28^ Adjustments were made for parity, maternal BMI, maternal age, pre-eclampsia, gestational age category and method of birth. Statistical significance was set at two-sided 5% level for all tests. Reporting of this study adheres to the Strengthening the Reporting of Observational Studies in Epidemiology (STROBE) statement.^29^

### Ethics committee approval

We utilized the Queensland Perinatal Data Collection^18^ with institutional ethical approval granted by the Metro North Hospital and Health Service Human Research Ethics Committee (Reference number: LNR/219/QRBC/53154).

### Role of the funding source

Prof Kumar is supported by the Mater Foundation and receives research funding from the Australian Medical Research Future Fund and the National Health and Medical Research Council. Jesrine Hong is supported by a University of Queensland Research Scholarship and the Mater Foundation. None of the funding parties had any role in the design and conduct of the study; collection, management, analysis, and interpretation of the data; preparation, review, or approval of the manuscript; and decision to submit the manuscript for publication.

## Results

**Fig. 1:**
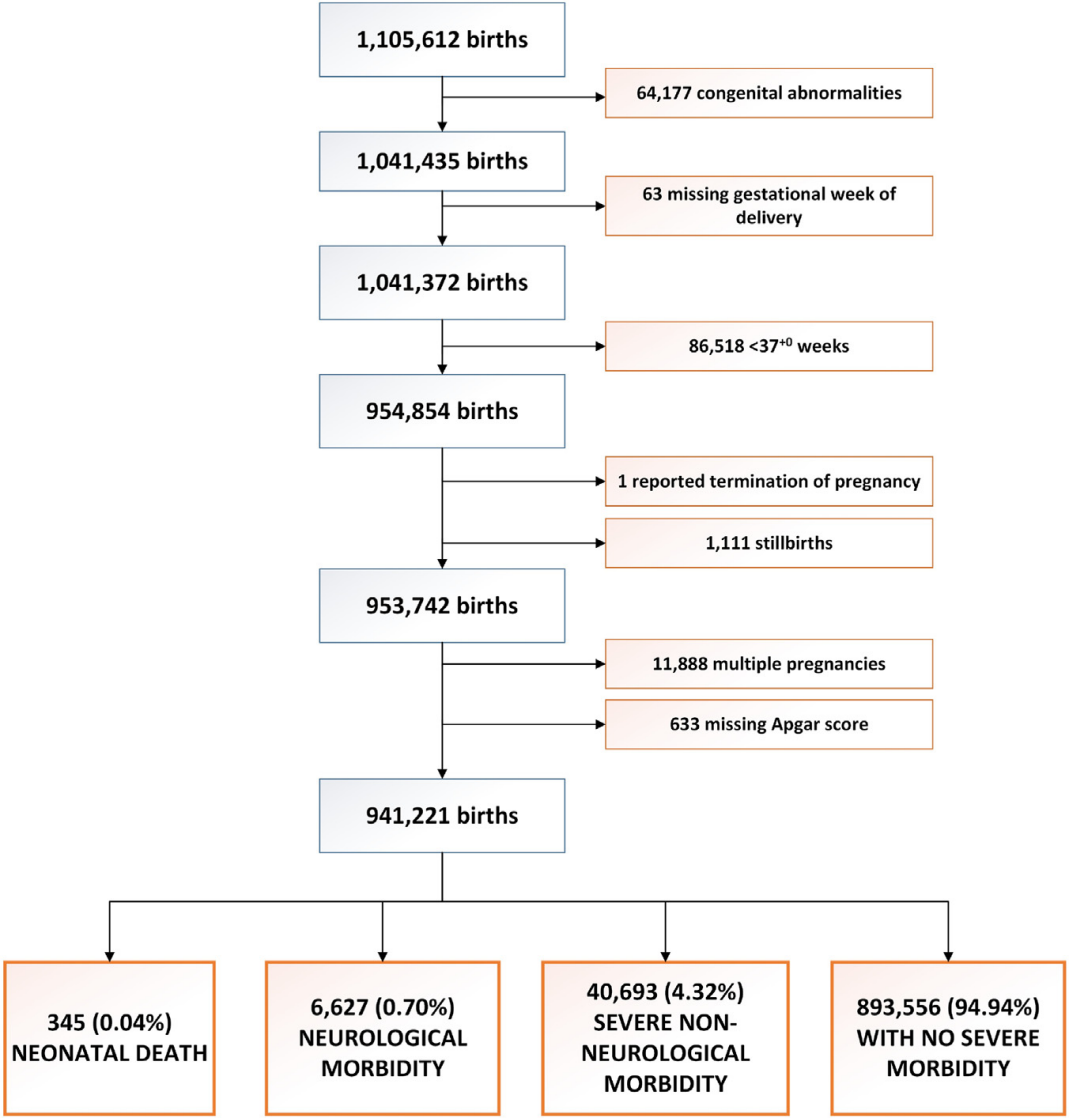
Flow diagram of inclusion, exclusion criteria and birth outcomes.

Of the initial dataset of 1,105,612 births, there were 941,221 term singleton births that met the inclusion criteria and were included in the final analysis (Fig. 1). Of these, 0.04% (345/941,221) were neonatal deaths, 0.70% (6627/941,221) were infants with severe neurological morbidity and 4.3% (40,693/941,221) had severe non-neurological morbidity.

### Maternal demographic characteristics and intrapartum outcomes

Table 1 details the unadjusted maternal demographic characteristics and intrapartum outcomes of the study population stratified by Apgar score categories. The majority (98.9%, 931,492/941,221) of the study cohort had an Apgar score of >7. Women who birthed infants with an Apgar score of <7 had higher relative rates of nulliparity, Indigenous ethnicity, previous miscarriage <20 weeks' gestation, antepartum hemorrhage >20 weeks’ gestation, smoking, use of illicit drugs, BMI ≥35 kg/m^2^, diabetes mellitus, pre-eclampsia and were in the lowest socio-economic quintile. These women were also more likely to birth at home or, have induction of labor, experience unsuccessful induction of labor, have longer duration of labor, higher relative rates of instrumental vaginal birth and emergency caesarean section. Adverse intrapartum events such as non-reassuring fetal status, cord prolapse, intrapartum hemorrhage, uterine rupture, and failed instrumental delivery were also higher in women who birthed infants with Apgar score <4 at 5-min.

### Neonatal outcomes

Table 2 lists the unadjusted study outcomes stratified by Apgar score categories. Infants with Apgar score <4 were more likely to birth at late term and post term gestations and have birthweights <3rd and <10th percentile for gestation. Rates of neonatal mortality increased with decreasing Apgar scores with infants in the Apgar score 0 or 1 group having the highest rates compared to those with a score of 9 or 10 (23.2% vs. 0.0%). Infants with an Apgar score of 0 or 1 also had the highest rates of birth trauma (10.6%). Infants in Apgar score categories 0 or 1 and 2 or 3 had the highest rates of severe neurological morbidity (26.3% and 29.5% respectively). For infants with severe non-neurological morbidity, 10.6%, 19.6% and 22.9% had Apgar scores in the 0 or 1, 2 or 3 and 4–6 categories respectively compared to only 3.6% in the referent cohort.

**Table 2:** Neonatal outcomes stratified by Apgar score.

Apgar Score	Total	0 or 1	2 or 3	4 to 6	7 or 8	9 or 10
	N = 941,221	N = 388	N = 959	N = 8382	N = 43,587	N = 887,905
Gestational age (completed weeks)	39 (38, 40)	40 (38, 40)	40 (38, 40)	39 (38, 40)	39 (38, 40)	39 (38, 40)
Gestational age categories						
Early term (37^+0^–38^+6^ weeks)	267,877 (28.5%)	108 (27.8%)	283 (29.5%)	2411 (28.8%)	12,741 (29.2%)	252,334 (28.4%)
Full term (39^+0^–40^+6^ weeks)	537,755 (57.1%)	195 (50.3%)	465 (48.5%)	4323 (51.6%)	23,033 (52.8%)	509,739 (57.4%)
Late term (41^+0^–41^+6^ weeks)	128,336 (13.6%)	75 (19.3%)	199 (20.8%)	1549 (18.5%)	7301 (16.8%)	119,212 (13.4%)
Post term (>42 weeks)	7253 (0.8%)	10 (2.6%)	12 (1.3%)	99 (1.2%)	512 (1.2%)	6620 (0.7%)
Birthweight (g)	3470 (3170, 3782)	3452 (3060, 3846)	3450 (3110, 3760)	3440 (3115, 3790)	3475 (3150, 3815)	3470 (3170, 3780)
Birthweight <3rd centile	22,190 (2.4%)	20 (5.2%)	49 (5.1%)	415 (5.0%)	1443 (3.3%)	20,263 (2.3%)
Birthweight <10th centile	79,045 (8.4%)	53 (13.8%)	123 (12.9%)	1035 (12.4%)	4497 (10.3%)	73,337 (8.3%)
***Severe adverse neonatal outcome***						
Death within 28 days	345 (0.0%)	90 (23.2%)	49 (5.1%)	47 (0.6%)	30 (0.1%)	129 (0.0%)
Severe neurological morbidity^a^	6627 (0.7%)	102 (26.3%)	283 (29.5%)	1604 (19.1%)	2118 (4.9%)	2520 (0.3%)
Severe non-neurological morbidity^b^	40,693 (4.3%)	41 (10.6%)	188 (19.6%)	1917 (22.9%)	6384 (14.6%)	32,163 (3.6%)
No severe morbidity	893,556 (94.9%)	155 (39.9%)	439 (45.8%)	4814 (57.4%)	35,055 (80.4%)	853,093 (96.1%)
***Neonatal adverse outcome***						
Birth asphyxia	6798 (0.7%)	187 (48.2%)	328 (34.2%)	1639 (19.6%)	2128 (4.9%)	2516 (0.3%)
Hypoxic ischemic encephalopathy	533 (0.1%)	108 (27.8%)	93 (9.7%)	196 (2.3%)	91 (0.2%)	45 (0.0%)
Neonatal seizures	740 (0.1%)	26 (6.7%)	57 (5.9%)	144 (1.7%)	137 (0.3%)	376 (0.0%)
Intraventricular hemorrhage	98 (0.0%)	2 (0.5%)	1 (0.1%)	10 (0.1%)	22 (0.1%)	63 (0.0%)
Acidosis at birth (Cord Artery pH <7.0)	1433 (1.1%)	39 (33.1%)	68 (19.3%)	321 (10.3%)	478 (4.1%)	527 (0.5%)
Severe resuscitation required^c^	20,235 (2.1%)	240 (61.9%)	533 (55.6%)	2267 (27.0%)	4246 (9.7%)	12,949 (1.5%)
Admission >24 h	6556 (0.7%)	62 (16.0%)	151 (15.7%)	1030 (12.3%)	2061 (4.7%)	3252 (0.4%)
Neonatal sepsis^d^	40,191 (4.3%)	125 (32.2%)	304 (31.7%)	2315 (27.6%)	6386 (14.7%)	31,061 (3.5%)
Birth trauma^e^	6773 (0.7%)	41 (10.6%)	35 (3.6%)	273 (3.3%)	989 (2.3%)	5435 (0.6%)
Necrotizing enterocolitis	27 (0.0%)	1 (0.3%)	2 (0.2%)	1 (0.0%)	3 (0.0%)	20 (0.0%)
Hypoglycemia	24,383 (2.6%)	43 (11.1%)	109 (11.4%)	896 (10.7%)	2879 (6.6%)	20,456 (2.3%)
Hypothermia	7773 (0.8%)	23 (5.9%)	23 (2.4%)	220 (2.6%)	831 (1.9%)	6676 (0.8%)
Shoulder dystocia	9728 (1.0%)	28 (7.2%)	34 (3.5%)	453 (5.4%)	1773 (4.1%)	7440 (0.8%)

Data are presented as median (IQR) for continuous measures, and (N) column % for categorical measures. NICU – Neonatal Intensive Care Unit.

a Severe neurological morbidity included birth asphyxia, neonatal encephalopathy, neonatal seizures and intraventricular hemorrhage.

b Severe non-neurological morbidity included neonatal intensive care unit (NICU) admission for >24 h, neonatal sepsis or necrotizing enterocolitis.

c Severe resuscitation required included neonates requiring intermittent positive pressure ventilation via an endotracheal tube, external cardiac massage, administration of ionotropic or chronotropic drugs, or sodium bicarbonate.

d Neonatal sepsis included bacterial, viral and fungal sepsis.

e Birth trauma included extracranial hemorrhage, scalp injuries, facial or eye trauma, injury to the facial or peripheral nerves, fractures (skull, facial, clavicular or long bones), or intra-abdominal trauma.

### Rate differences for study outcomes

**Fig. 2:**
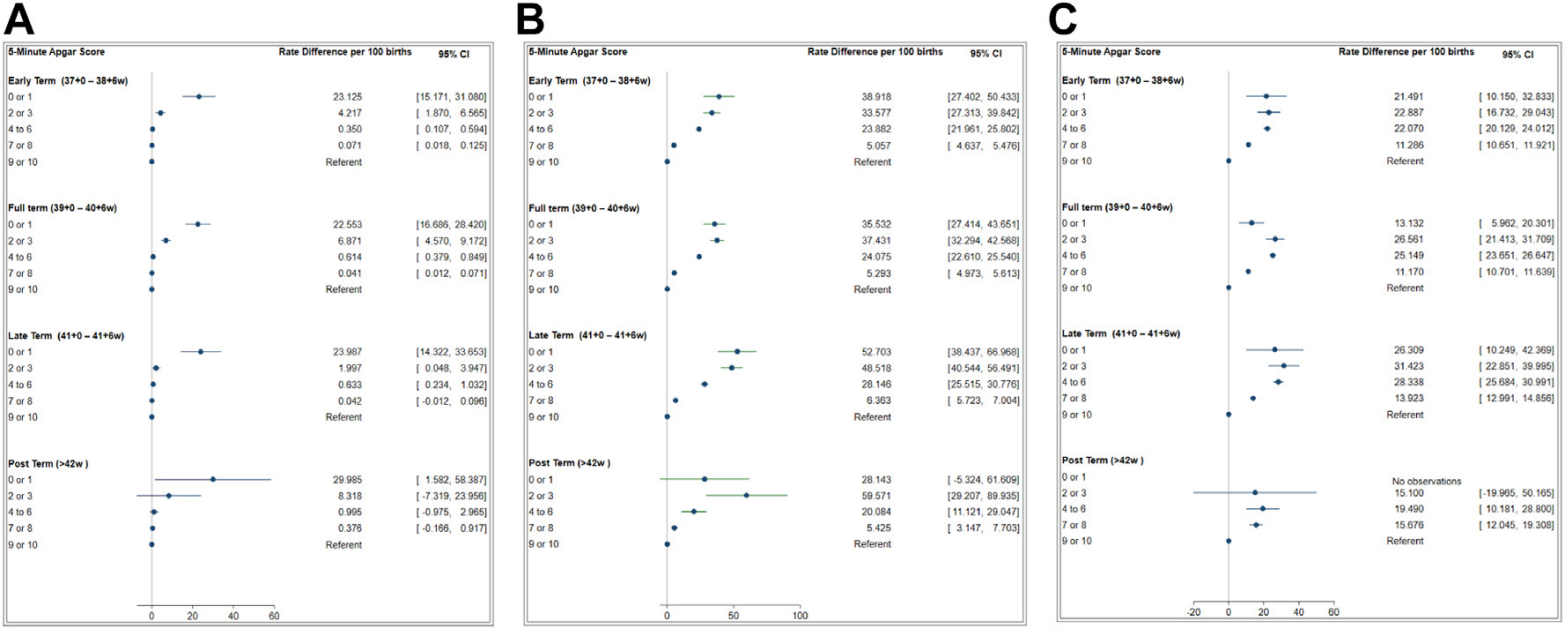
Rate differences in A) Neonatal mortality B) Severe neurological morbidity and C) Severe non-neurological morbidity per 100 births.

Fig. 2 demonstrates the rate difference per 100 births (i.e., excess number of adverse outcomes per 100 births) for the three study outcomes. For neonatal mortality, the absolute rate difference was significantly greater in the Apgar 0 or 1 group compared to the referent cohort (Apgar 9 or 10) for all term gestational age categories. The rate difference ranged from 23.13 (95% CI 15.17–31.08) in the early term cohort to 29.99 (1.58–53.89) in the post term group. For severe neurological and non-neurologic morbidity, the rate differences increased for all other Apgar scores categories compared to the referent group with the highest rate difference seen in the late term cohort.

### Adjusted relative risk ratios for study outcomes

Table 3 details the Poisson multivariable analyses for the study outcomes stratified by term gestational age and Apgar score categories. Goodness of fit tests indicated that data were not over-dispersed for all outcomes analyzed (p > 0.05). Regardless of gestational age category, Apgar scores of 0 or 1, 2 or 3, 4–6 and 7 or 8 were all strongly associated with neonatal mortality, severe neurological morbidity and severe non-neurologic morbidity. For all three study outcomes, the adjusted relative risks were highest in the Apgar 0 or 1 category. For neonatal mortality, the highest aRRR was seen in post term infants that had an Apgar score of 0 or 1 (aRRR 2231.59, 95% CI 272.23, 18293.07, p < 0.001). In contrast severe neurological morbidity was highest in early term infants with an Apgar score of 0 or 1 (aRRR 158.48, 95% CI 118.74, 211.51, p < 0.001) whilst severe non-neurological morbidity was greatest in the full term, Apgar 2–3 cohort (aRRR 7.36, 95% CI 6.2, 8.74, p < 0.001). Alternative confounders were also investigated revealing similar results (Supplementary Tables S1 and S2).

**Table 3:** Multivariable analyses of the effect of 5-min Apgar score on neonatal outcomes, stratified by gestational age.

Total population	Total	Neonatal death					Neurological morbidity					Non-neurologic morbidity				
	Total infants N = 941,221	N = 345	Univariable RRR (95% CI)	p-value	Adjusted RRR (95% CI)	p-value	N = 6627	Univariable RRR (95% CI)	p-value	Adjusted RRR (95% CI)	p-value	N = 40,693	Univariable RRR (95% CI)	p-value	Adjusted RRR (95% CI)	p-value
**5-min Apgar score**																
0 or 1	388 (0.04)	90 (26.09)	1590.24 (1238.28, 2042.23)	<0.001	1303.60 (1009.87, 1682.76)	<0.001	102 (1.54)	134.09 (114.78, 156.65)	<0.001	111.59 (96.19, 129.46)	<0.001	41 (0.10)	5.74 (4.37, 7.54)	<0.001	5.00 (3.83, 6.53)	<0.001
2 or 3	959 (0.10)	49 (14.20)	350.67 (253.93, 484.27)	<0.001	288.56 (207.53, 401.24)	<0.001	283 (4.27)	132.72 (120.26, 146.48)	<0.001	110.63 (100.14, 122.21)	<0.001	188 (0.46)	8.24 (7.31, 9.29)	<0.001	6.89 (6.11, 7.78)	<0.001
4–6	8382 (0.89)	47 (13.62)	38.52 (27.6, 53.76)	<0.001	33.06 (23.46, 46.60)	<0.001	1604 (24.20)	84.75 (80.01, 89.77)	<0.001	69.86 (65.74, 74.23)	<0.001	1917 (4.71)	7.83 (7.52, 8.14)	<0.001	6.18 (5.94, 6.44)	<0.001
7 or 8	43,587 (4.63)	30 (8.70)	4.73 (3.18, 7.04)	<0.001	4.49 (3.03, 6.65)	<0.001	2118 (31.96)	19.33 (18.26, 20.46)	<0.001	16.97 (16, 17.99)	<0.001	6384 (15.69)	4.24 (4.13, 4.34)	<0.001	3.51 (3.42, 3.6)	<0.001
9 or 10	887,905 (94.34)	129 (37.39)	Referent		Referent		2520 (38.03)	Referent		Referent		32,163 (79.04)	Referent		Referent	
**Early Term (37**^**+0**^–**38**^**+6**^**wks)**	**N** = **267,877**	**N** = **116**	**Univariable RRR (95% CI)**	**p-value**	**Adjusted RRR(95% CI)**	**p-value**	**N** = **1654**	**Univariable RRR (95% CI)**	**p-value**	**Adjusted RRR(95% CI)**	**p-value**	**N** = **11,194**	**Univariable RRR (95% CI)**	**p-value**	**Adjusted RRR(95% CI)**	**p-value**
**5-min Apgar score**																
0 or 1	108 (0.04)	25 (21.55)	1009.81 (657.37, 1551.2)	<0.001	860.16 (560.96, 1318.94)	<0.001	27 (1.63)	183.90 (135.34, 249.88)	<0.001	158.48 (118.74, 211.51)	<0.001	14 (0.13)	7.15 (4.53, 11.27)	<0.001	6.42 (4.16, 9.93)	<0.001
2 or 3	283 (0.11)	12 (10.34)	184.91 (100.4, 340.53)	<0.001	166.51 (90.41, 306.68)	<0.001	74 (4.47)	158.80 (129.45, 194.81)	<0.001	140.65 (114.84, 172.26)	<0.001	52 (0.46)	7.54 (5.97, 9.53)	<0.001	6.88 (5.45, 8.68)	<0.001
4–6	2411 (0.90)	9 (7.76)	16.29 (8.08, 32.84)	<0.001	14.74 (7.17, 30.29)	<0.001	459 (27.75)	113.24 (100.71, 127.33)	<0.001	98.26 (86.99, 110.99)	<0.001	497 (4.44)	7.31 (6.76, 7.91)	<0.001	6.19 (5.71, 6.7)	<0.001
7 or 8	12,741 (4.76)	12 (10.34)	4.11 (2.21, 7.65)	<0.001	3.91 (2.11, 7.23)	<0.001	576 (34.82)	24.76 (22.03, 27.84)	<0.001	22.63 (20.09, 25.48)	<0.001	1798 (16.06)	4.23 (4.03, 4.43)	<0.001	3.71 (3.54, 3.9)	<0.001
9 or 10	252,334 (94.20)	58 (50.00)	Referent				518 (31.32)	Referent				8833 (78.91)	Referent			
**Full term (39**^**+0**^–**40**^**+6**^**wks)**	**N** = **537,755**	**N** = **170**	**Univariable RRR (95% CI)**	**p-value**	**Adjusted RRR(95% CI)**	**p-value**	**N** = **3509**	**Univariable RRR (95% CI)**	**p-value**	**Adjusted RRR(95% CI)**	**p-value**	**N** = **21,436**	**Univariable RRR (95% CI)**	**p-value**	**Adjusted RRR(95% CI)**	**p-value**
**5-min Apgar score**																
0 or 1	195 (0.04)	44 (25.88)	2087.46 (1440.61, 3024.76)	<0.001	1835.77 (1279.48, 2633.91)	<0.001	48 (1.37)	123.85 (98.18, 156.23)	<0.001	112.99 (90.56, 140.98)	<0.001	17 (0.08)	4.88 (3.16, 7.54)	<0.001	4.63 (3.03, 7.08)	<0.001
2 or 3	465 (0.09)	32 (18.82)	636.57 (415.69, 974.82)	<0.001	551.35 (363.77, 835.66)	<0.001	129 (3.68)	130.52 (112.82, 150.99)	<0.001	112.9 (97.67, 130.52)	<0.001	91 (0.42)	8.86 (7.46, 10.53)	<0.001	7.36 (6.2, 8.74)	<0.001
4–6	4323 (0.80)	27 (15.88)	57.81 (36.51, 91.53)	<0.001	52.61 (33.46, 82.7)	<0.001	804 (22.91)	84.34 (77.9, 91.31)	<0.001	71.86 (66.19, 78.01)	<0.001	996 (4.65)	8.44 (8, 8.92)	<0.001	6.73 (6.36, 7.11)	<0.001
7 or 8	23,033 (4.28)	12 (7.06)	4.82 (2.58, 9.01)	<0.001	4.68 (2.54, 8.63)	<0.001	1107 (31.55)	19.33 (17.89, 20.88)	<0.001	17.3 (15.98, 18.72)	<0.001	3187 (14.87)	4.31 (4.16, 4.46)	<0.001	3.58 (3.45, 3.71)	<0.001
9 or 10	509,739 (94.79)	55 (32.35)	Referent				1421 (40.50)	Referent				17,145 (79.98)	Referent			
**Late term (41**^**+0**^–**41**^**+6**^**wks)**	**N** = **128,336**	**N** = **51**	**Univariable RRR (95% CI)**	**p-value**	**Adjusted RRR(95% CI)**	**p-value**	**N** = **1389**	**Univariable RRR (95% CI)**	**p-value**	**Adjusted RRR(95% CI)**	**p-value**	**N** = **7619**	**Univariable RRR (95% CI)**	**p-value**	**Adjusted RRR(95% CI)**	**p-value**
**5-min Apgar score**																
0 or 1	75 (0.06)	18 (35.29)	1907.39 (999, 3641.77)	<0.001	1693.61 (859.65, 3336.6)	<0.001	25 (1.80)	108.82 (82.18, 144.09)	<0.001	87.94 (67.09, 115.27)	<0.001	10 (0.13)	6.32 (3.78, 10.58)	<0.001	4.92 (3.08, 7.87)	<0.001
2 or 3	199 (0.16)	4 (7.84)	159.75 (53.49, 477.1)	<0.001	149.15 (49.31, 451.15)	<0.001	74 (5.33)	100.26 (83.52, 120.35)	<0.001	84.1 (69.82, 101.3)	<0.001	44 (0.58)	7.36 (5.81, 9.33)	<0.001	6.11 (4.77, 7.81)	<0.001
4–6	1549 (1.21)	10 (19.61)	51.31 (23.09, 114.02)	<0.001	49.43 (22.05, 110.79)	<0.001	325 (23.40)	58.58 (51.76, 66.3)	<0.001	50.71 (44.63, 57.61)	<0.001	404 (5.30)	6.74 (6.2, 7.32)	<0.001	5.47 (5.03, 5.96)	<0.001
7 or 8	7301 (5.69)	4 (7.84)	4.35 (1.45, 13.12)	0.009	4.31 (1.41, 13.18)	0.01	411 (29.59)	14.02 (12.37, 15.88)	<0.001	12.57 (11.07, 14.26)	<0.001	1299 (17.05)	3.82 (3.61, 4.03)	<0.001	3.17 (3, 3.35)	<0.001
9 or 10	119,212 (92.89)	15 (29.41)	Referent		Referent		554 (39.88)	Referent		Referent		5862 (76.94)	Referent		Referent	
**Post term (>42 wks)**	**N** = **7253**	**N** = **8**	**Univariable RRR (95% CI)**	**p-value**	**Adjusted RRR(95% CI)**	**p-value**	**N** = **75**	**Univariable RRR (95% CI)**	**p-value**	**Adjusted RRR(95% CI)**	**p-value**	**N** = **444**	**Univariable RRR (95% CI)**	**p-value**	**Adjusted RRR(95% CI)**	**p-value**
**5-min Apgar score**																
0 or 1	10 (0.14)	3 (37.50)	1977.37 (224.08, 17448.89)	<0.001	2231.59 (272.23, 18293.07)	<0.001	2 (2.67)	66.2 (19.39, 226.06)	<0.001	52.07 (15.17, 178.7)	<0.001	0 (0.00)	#	#	#	#
2 or 3	12 (0.17)	1 (12.50)	551.88 (36.59, 8323.73)	<0.001	432.05 (30.88, 6045.85)	<0.001	6 (8.00)	139.97 (74.49, 262.99)	<0.001	137.09 (77.46, 242.62)	<0.001	1 (0.23)	4.08 (0.71, 23.65)	0.116	6.74 (1.12, 40.58)	0.037
4–6	99 (1.36)	1 (12.50)	66.86 (4.21, 1061.6)	0.003	74.73 (4.62, 1209.71)	0.002	16 (21.33)	47.84 (26.87, 85.16)	<0.001	39.17 (21.39, 71.74)	<0.001	20 (4.50)	4.98 (3.35, 7.39)	<0.001	4.31 (2.82, 6.59)	<0.001
7 or 8	512 (7.06)	2 (25.00)	25.86 (2.35, 284.72)	0.008	28.8 (2.84, 292.34)	0.004	24 (32.00)	13.65 (7.95, 23.44)	<0.001	11.49 (6.55, 20.16)	<0.001	100 (22.52)	4.2 (3.42, 5.15)	<0.001	3.67 (2.97, 4.54)	<0.001
9 or 10	6620 (91.27)	1 (12.50)	Referent		Referent		27 (36.00)	Referent		Referent		323 (72.75)	Referent		Referent	

N total number (column %); RRR = Incidence Rate Ratio; # = No observations.

## Discussion

In this cohort study of term infants from Queensland, Australia, we found that 5-min Apgar scores were inversely related with the risk of neonatal death, severe neurological morbidity and severe non-neurologic morbidity across all term gestational age categories. We demonstrate that the relative risks for all three study outcomes were incrementally greater in infants with Apgar scores <9 compared with Apgar scores 9 or 10. Our results also show that the relative risks were highest in infants with Apgar scores 0 or 1–for neonatal mortality the post-term gestational age group had the greatest relative risk whilst for severe neurological morbidity they were greatest in the early term cohort. However, for severe non-neurological morbidity, the risks were broadly similar for all Apgar score groups regardless of gestational age, with the highest risk present when the Apgar score was <4.

Our results indicate that nulliparity, Indigenous status, antepartum hemorrhage, smoking, illicit drug use, raised BMI, low maternal socioeconomic status, diabetes mellitus, pre-eclampsia, induction of labor, intrapartum non-reassuring fetal status, cord prolapse, intrapartum hemorrhage, uterine rupture and operative delivery are all significantly associated with low 5-min Apgar scores. Our findings and that of others support the view that an Apgar <4 at 5-min score is a useful indicator of the probability of neonatal death, severe neurological morbidity in term infants as it is in preterm infants.^19^^,^^30^

Our findings are consistent with that of Iliodromiti et al.^6^ who, in an analysis of Scottish term and preterm births demonstrated that an Apgar score <4 was strongly associated with neonatal death (adjusted relative risks (aRR) 359.4, 95% CI 277.3, 465.9) for early neonatal and late neonatal death (aRR 30.5, 95% CI 18.0–51.6). The strongest associations for mortality were attributable to birth asphyxia and Apgar score <4 for term infants (RR 961.7, 95% CI 681.3, 357.5). Indeed, our findings of severe neurological morbidity not only concur with Iliodromiti et al.*'s*^6^ results but also provide additional detail by demonstrating that the risks of brain injury potentially attributable to birth asphyxia are greatest in the early term cohort. The early term brain, although relatively mature, is smaller and more vulnerable than those of later gestations^31^–early term birth is associated with poorer long-term neurodevelopmental outcomes including cerebral palsy (aOR 1.75, 95% CI 1.32–3.31, p < 0.001) and intellectual impairment.^16^ In another study, the risk of neonatal mortality was increased ∼300 times in peri-viable infants born at 22–24 weeks' gestation compared to late preterm infants with similar 5-min Apgar scores of 0 or 1.^19^ In our study of term infants, the relative risk of neonatal mortality in infants with Apgar score 0 or 1 increased by almost three times from early term to post term gestational age whilst severe neurological morbidity decreased by a factor of three in post term compared to early term infants with Apgar score of 0 or 1.

Our findings of increased risks of neonatal mortality and severe neurological morbidity concur with other studies^3^^,^^9^^,^^32^^,^^33^ that show an Apgar of <7 at 5 min is associated with increased risks of neonatal and infant mortality, hypoxic-ischemic encephalopathy and longer term neurologic morbidity. We also found similar findings to Razaz et al.^7^ who reported that term infants with 5-min Apgar scores of 7–9 had higher risks of neonatal mortality and morbidity including asphyxia related complications compared to those with a score of 10. Our results extend these previous findings by demonstrating that 5-min Apgar scores are also strongly correlated with adverse neonatal outcomes when stratified by gestational age at term. We found that the relative risk of severe neurological morbidity was incrementally greatest in early term infants with Apgar scores <9 when compared with those with a score of 9 or 10. Our results from a different population provides further support that the risks of neonatal neurological morbidity are inversely associated with 5-min Apgar scores.^32^ Further evidence reflective of the clinical value of the Apgar score is also provided by a large population-based Swedish cohort study^34^ showing that, after adjusting for confounders, the likelihood of requiring therapeutic hypothermia was highest (12-fold increased odds) for infants with an Apgar score ≤5 at 10 min and those needing resuscitation at birth (9-fold increased odds). Nevertheless, the association between low 5-min Apgar score and neurological abnormalities as described in our study needs to be viewed through the prism of gestational age—other large cohort studies^35^ in preterm infants did not reveal an association between low Apgar scores and severe neurologic injury (defined as grade 3/4 intraventricular hemorrhage or cystic periventricular leukomalacia).

As the Apgar score is influenced by many extraneous factors including gestational age, the American Association of Pediatrics currently does not recommend its use to determine treatment decisions.^1^ The rationale for this view is reinforced by a recent study^36^ showing that even a 10-min Apgar score of zero did not predict either neonatal mortality or moderate/severe disability (area under the receiver operating characteristic curve of only 0.56) and suggests that resuscitative efforts should therefore continue beyond 10 min for these apparently moribund infants.

### Strengths and limitations

One of the strengths of our study is its size of ∼1 million births from a diverse ethnic and socioeconomic population thus increasing the generalizability of our results. Another strength is our inclusion of neonatal morbidity including severe neurological and non-neurological morbidity as co-primary outcomes. Previous studies^6^^,^^37^^,^^38^ evaluating Apgar scores among term infants have focused mainly on neonatal and infant mortality despite the fact that neonatal morbidity occurs at a much higher rate. We also attempted to provide more granularity of information by stratifying both Apgar scores and by term gestational age categories in an attempt to show the combined effect of both on outcomes.

Limitations of our study include its retrospective nature with potential errors in assigning ICD-10 codes, missing information and the low numbers of infants and adverse outcomes in the post-term cohort. Our dataset also lacked information on Apgar scores at 10-min. We also did not have detailed information on interventions provided during resuscitation and the scores of each component which may have influenced the overall 5-min score. This may be relevant because another recent study^39^ of term infants showed that metabolic acidosis at birth was associated with increased risks of reduced 5 and 10-min Apgar scores within the normal range (7–9) and the presence of metabolic acidosis and Apgar scores at 5 min (7 or 8 vs. 9 or 10) increased the risks of neonatal morbidity. We also acknowledge that the cohort of infants with severe non-neurologic morbidity will be underrepresented as all outcomes were considered mutually exclusive by severity ranking. Finally, we were also not able to ascertain if a woman's socio-economic status changed over the study period.

### Conclusion

Our study suggests that in term infants, low 5-min Apgar scores particularly <4, are strongly associated with neonatal mortality, severe neurological morbidity and severe non-neurologic morbidity with the greatest risk seen in early term infants.

## Contributors

SK conceived the study. KC compiled the database and analyzed the data. JH, KJ, TT, and SK analyzed the findings. JH prepared the first draft with KC contributed to the statistical analysis section. SK extensively revised the manuscript with input from all authors. All authors had access to and verified all data in the study, contributed to writing of the manuscript, and had final responsibility to submit for publication.

## Data sharing statement

All code, scripts and data used to produce the results in this article will be available to any researcher provided appropriate ethics approval, inter-institutional data sharing agreements and other regulatory requirements are in place. Additional specific approval from the Data Custodian of the Statistical Services Branch of Queensland's Department of Health will also be required.

## Declaration of interests

None.
