# Supplementary material for: Five-minute Apgar score and risk of neonatal mortality, severe neurological morbidity and severe non-neurological morbidity in term infants – an Australian population-based cohort study
Source: Lancet Reg Health West Pac. 2024 Jan 13;44:101011. doi: 10.1016/j.lanwpc.2024.101011 (PMC10825608; doi:10.1016/j.lanwpc.2024.101011)
Supplement: Supplementary Tables S1 and S2 [file mmc1.docx]

**Supplementary Table 1** Multivariable analyses of the effect of 5-minute Apgar score on neonatal outcomes, stratified by gestational age and adjusted for age and method of birth.

|  | **Total** | **Neonatal Death** | | | | | **Neurological Morbidity** | | | | | **Non-Neurologic Morbidity** | | | | |
| --- | --- | --- | --- | --- | --- | --- | --- | --- | --- | --- | --- | --- | --- | --- | --- | --- |
| **Early Term (37^+0^ – 38^+6^wks)** | **N=267877** | **N=116** | **Univariable RRR (95% CI)** | **p-value** | **Adjusted RRR^a^ (95% CI)** | **p-value** | **N=1654** | **Univariable RRR (95% CI)** | **p-value** | **Adjusted RRR^a^ (95% CI)** | **p-value** | **N=11194** | **Univariable RRR (95% CI)** | **p-value** | **Adjusted RRR^a^ (95% CI)** | **p-value** |
| **5-minute Apgar** |  |  |  |  |  |  |  |  |  |  |  |  |  |  |  |  |
| 0 or 1 | 108(0.04) | 25 (21.55) | 1009.81 (657.37, 1551.2) | <0.001 | 877.79 (571.22, 1348.9) | <0.001 | 27 (1.63) | 183.90 (135.34, 249.88) | <0.001 | 157.13 (117.36, 210.39) | <0.001 | 14 (0.13) | 7.15 (4.53, 11.27) | <0.001 | 6.36 (4.09, 9.89) | <0.001 |
| 2 or 3 | 283 (0.11) | 12 (10.34) | 184.91 (100.4, 340.53) | <0.001 | 159.84 (86.46, 295.49) | <0.001 | 74 (4.47) | 158.80 (129.45, 194.81) | <0.001 | 139.49 (113.89, 170.84) | <0.001 | 52 (0.46) | 7.54 (5.97, 9.53) | <0.001 | 6.88 (5.45, 8.7) | <0.001 |
| 4 to 6 | 2411 (0.90) | 9 (7.76) | 16.29 (8.08, 32.84) | <0.001 | 14.23 (6.93, 29.2) | <0.001 | 459 (27.75) | 113.24 (100.71, 127.33) | <0.001 | 97.6 (86.37, 110.3) | <0.001 | 497 (4.44) | 7.31 (6.76, 7.91) | <0.001 | 6.21 (5.74, 6.73) | <0.001 |
| 7 or 8 | 12741 (4.76) | 12 (10.34) | 4.11 (2.21, 7.65) | <0.001 | 3.83 (2.07, 7.1) | <0.001 | 576 (34.82) | 24.76 (22.03, 27.84) | <0.001 | 22.55 (20.02, 25.39) | <0.001 | 1798 (16.06) | 4.23 (4.03, 4.43) | <0.001 | 3.72 (3.54, 3.9) | <0.001 |
| 9 or 10 | 252334 (94.20) | 58 (50.00 | Referent |  | Referent |  | 518 (31.32) | Referent |  | Referent |  | 8833 (78.91) | Referent |  | Referent |  |
| **Full term (39^+0^ – 40^+6^wks)** | **N=537755** | **N=170** | **Univariable RRR (95% CI)** | **p-value** | **Adjusted RRR^a^ (95% CI)** | **p-value** | **N=3509** | **Univariable RRR (95% CI)** | **p-value** | **Adjusted RRR^a^ (95% CI)** | **p-value** | **N=21436** | **Univariable RRR (95% CI)** | **p-value** | **Adjusted RRR^a^ (95% CI)** | **p-value** |
| **5-minute Apgar** |  |  |  |  |  |  |  |  |  |  |  |  |  |  |  |  |
| 0 or 1 | 195 (0.04) | 44 (25.88) | 2087.46 (1440.61, 3024.76) | <0.001 | 1816.99 (1262.81, 2614.36) | <0.001 | 48 (1.37) | 123.85 (98.18, 156.23) | <0.001 | 112.74 (90.33, 140.72) | <0.001 | 17 (0.08) | 4.88 (3.16, 7.54) | <0.001 | 4.69 (3.07, 7.15) | <0.001 |
| 2 or 3 | 465 (0.09) | 32 (18.82) | 636.57 (415.69, 974.82) | <0.001 | 530.25 (347.1, 810.05) | <0.001 | 129 (3.68) | 130.52 (112.82, 150.99) | <0.001 | 112.14 (96.92, 129.76) | <0.001 | 91 (0.42) | 8.86 (7.46, 10.53) | <0.001 | 7.44 (6.28, 8.83) | <0.001 |
| 4 to 6 | 4323 (0.80) | 27 (15.88) | 57.81 (36.51, 91.53) | <0.001 | 51.12 (32.28, 80.94) | <0.001 | 804 (22.91) | 84.34 (77.9, 91.31) | <0.001 | 71.56 (65.9, 77.72) | <0.001 | 996 (4.65) | 8.44 (8, 8.92) | <0.001 | 6.82 (6.45, 7.21) | <0.001 |
| 7 or 8 | 23033 (4.28) | 12 (7.06) | 4.82 (2.58, 9.01) | <0.001 | 4.58 (2.47, 8.47) | <0.001 | 1107 (31.55) | 19.33 (17.89, 20.88) | <0.001 | 17.26 (15.94, 18.68) | <0.001 | 3187 (14.87) | 4.31 (4.16, 4.46) | <0.001 | 3.63 (3.5, 3.76) | <0.001 |
| 9 or 10 | 509739 (94.79) | 55 (32.35) | Referent |  | Referent |  | 1421 (40.50) | Referent |  | Referent |  | 17145 (79.98) | Referent |  | Referent |  |
| **Late Term (41^+0^ – 41^+6^wks)** | **N=128336** | **N=51** | **Univariable RRR (95% CI)** | **p-value** | **Adjusted RRR^a^ (95% CI)** | **p-value** | **N=1389** | **Univariable RRR (95% CI)** | **p-value** | **Adjusted RRR^a^ (95% CI)** | **p-value** | **N=7619** | **Univariable RRR (95% CI)** | **p-value** | **Adjusted RRR^a^ (95% CI)** | **p-value** |
| **5-minute Apgar** |  |  |  |  |  |  |  |  |  |  |  |  |  |  |  |  |
| 0 or 1 | 75 (0.06) | 18 (35.29) | 1907.39 (999, 3641.77) | <0.001 | # |  | 25 (1.80) | 108.82 (82.18, 144.09) | <0.001 | 89.33 (67.86, 117.59) | <0.001 | 10 (0.13) | 6.32 (3.78, 10.58) | <0.001 | 4.91 (3.09, 7.81) | <0.001 |
| 2 or 3 | 199 (0.16) | 4 (7.84) | 159.75 (53.49, 477.1) | <0.001 | # |  | 74 (5.33) | 100.26 (83.52, 120.35) | <0.001 | 85.04 (70.33, 102.82) | <0.001 | 44 (0.58) | 7.36 (5.81, 9.33) | <0.001 | 6.17 (4.81, 7.9) | <0.001 |
| 4 to 6 | 1549 (1.21) | 10 (19.61) | 51.31 (23.09, 114.02) | <0.001 | ǂ |  | 325 (23.40) | 58.58 (51.76, 66.3) | <0.001 | 51.54 (45.33, 58.6) | <0.001 | 404 (5.30) | 6.74 (6.2, 7.32) | <0.001 | 5.62 (5.16, 6.12) | <0.001 |
| 7 or 8 | 7301 (5.69) | 4 (7.84) | 4.35 (1.45, 13.12) | 0.009 | ǂ |  | 411 (29.59) | 14.02 (12.37, 15.88) | <0.001 | 12.75 (11.23, 14.47) | <0.001 | 1299 (17.05) | 3.82 (3.61, 4.03) | <0.001 | 3.25 (3.07, 3.43) | <0.001 |
| 9 or 10 | 119212 (92.89) | 15 (29.41) | Referent |  |  |  | 554 (39.88) | Referent |  | Referent |  | 5862 (76.94) | Referent |  | Referent |  |
| **Post Term (>42wks)** | **N=7253** | **N=8** | **Univariable RRR (95% CI)** | **p-value** | **Adjusted RRR^a^ (95% CI)** | **p-value** | **N=75** | **Univariable RRR (95% CI)** | **p-value** | **Adjusted RRR^a^ (95% CI)** | **p-value** | **N=444** | **Univariable RRR (95% CI)** | **p-value** | **Adjusted RRR^a^ (95% CI)** | **p-value** |
| **5-minute Apgar** |  |  |  |  |  |  |  |  |  |  |  |  |  |  |  |  |
| 0 or 1 | 10 (0.14) | 3 (37.50) | 1977.37 (224.08, 17448.89) | <0.001 | # |  | 2 (2.67) | 66.2 (19.39, 226.06) | <0.001 | 54.02 (16.53, 176.59) | <0.001 | 0 (0.00) | # | # | # | # |
| 2 or 3 | 12 (0.17) | 1 (12.50) | 551.88 (36.59, 8323.73) | <0.001 | # |  | 6 (8.00) | 139.97 (74.49, 262.99) | <0.001 | 144.41 (79.36, 262.78) | <0.001 | 1 (0.23) | 4.08 (0.71, 23.65) | 0.116 | 6.33 (1.09, 36.82) | 0.04 |
| 4 to 6 | 99 (1.36) | 1 (12.50) | 66.86 (4.21, 1061.6) | 0.003 | # |  | 16 (21.33) | 47.84 (26.87, 85.16) | <0.001 | 39.29 (20.97, 73.61) | <0.001 | 20 (4.50) | 4.98 (3.35, 7.39) | <0.001 | 4.31 (2.82, 6.59) | <0.001 |
| 7 or 8 | 512 (7.06) | 2 (25.00) | 25.86 (2.35, 284.72) | 0.008 | ǂ |  | 24 (32.00) | 13.65 (7.95, 23.44) | <0.001 | 11.97 (6.85, 20.92) | <0.001 | 100 (22.52) | 4.2 (3.42, 5.15) | <0.001 | 3.78 (3.06, 4.66) | <0.001 |
| 9 or 10 | 6620 (91.27) | 1 (12.50) | Referent |  | Referent |  | 27 (36.00) | Referent |  | Referent |  | 323 (72.75) | Referent |  | Referent |  |

N total number (column %); RRR= Relative Risk Ratio; ^a^ Adjusted for age and method of birth; ^#^ no observations ǂ Model would not converge due to absence of observations in more than 1 subgroup.

**Supplementary Table 2** Multivariable analyses of the effect of 5-minute Apgar score on neonatal outcomes, stratified by gestational age and adjusted for preeclampsia and body mass index.

|  | **Total** | **Neonatal Death** | | | | | **Neurological Morbidity** | | | | | **Non-Neurologic Morbidity** | | | | |
| --- | --- | --- | --- | --- | --- | --- | --- | --- | --- | --- | --- | --- | --- | --- | --- | --- |
| **Early Term (37^+0^ – 38^+6^wks)** | **N=267877** | **N=116** | **Univariable RRR (95% CI)** | **p-value** | **Adjusted RRR^a^ (95% CI)** | **p-value** | **N=1654** | **Univariable RRR (95% CI)** | **p-value** | **Adjusted RRR^a^ (95% CI)** | **p-value** | **N=11194** | **Univariable RRR (95% CI)** | **p-value** | **Adjusted RRR^a^ (95% CI)** | **p-value** |
| **5 minute Apgar** |  |  |  |  |  |  |  |  |  |  |  |  |  |  |  |  |
| 0 or 1 | 108(0.04) | 25 (21.55) | 1009.81 (657.37, 1551.2) | <0.001 | 991.17 (646.63, 1519.29) | <0.001 | 27 (1.63) | 183.90 (135.34, 249.88) | <0.001 | # |  | 14 (0.13) | 7.15 (4.53, 11.27) | <0.001 | 7.21 (4.58, 11.34) | <0.001 |
| 2 or 3 | 283 (0.11) | 12 (10.34) | 184.91 (100.4, 340.53) | <0.001 | 180.5 (98.33, 331.34) | <0.001 | 74 (4.47) | 158.80 (129.45, 194.81) | <0.001 | # |  | 52 (0.46) | 7.54 (5.97, 9.53) | <0.001 | 7.39 (5.84, 9.34) | <0.001 |
| 4 to 6 | 2411 (0.90) | 9 (7.76) | 16.29 (8.08, 32.84) | <0.001 | 15.86 (7.81, 32.21) | <0.001 | 459 (27.75) | 113.24 (100.71, 127.33) | <0.001 | ǂ |  | 497 (4.44) | 7.31 (6.76, 7.91) | <0.001 | 7.12 (6.58, 7.71) | <0.001 |
| 7 or 8 | 12741 (4.76) | 12 (10.34) | 4.11 (2.21, 7.65) | <0.001 | 4.02 (2.17, 7.45) | <0.001 | 576 (34.82) | 24.76 (22.03, 27.84) | <0.001 | ǂ |  | 1798 (16.06) | 4.23 (4.03, 4.43) | <0.001 | 4.14 (3.94, 4.34) | <0.001 |
| 9 or 10 | 252334 (94.20) | 58 (50.00 | Referent |  | Referent |  | 518 (31.32) | Referent |  | Referent |  | 8833 (78.91) | Referent |  | Referent |  |
| **Full term (39^+0^ – 40^+6^wks)** | **N=537755** | **N=170** | **Univariable RRR (95% CI)** | **p-value** | **Adjusted RRR^a^ (95% CI)** | **p-value** | **N=3509** | **Univariable RRR (95% CI)** | **p-value** | **Adjusted RRR^a^ (95% CI)** | **p-value** | **N=21436** | **Univariable RRR (95% CI)** | **p-value** | **Adjusted RRR^a^ (95% CI)** | **p-value** |
| **5 minute Apgar** |  |  |  |  |  |  |  |  |  |  |  |  |  |  |  |  |
| 0 or 1 | 195 (0.04) | 44 (25.88) | 2087.46 (1440.61, 3024.76) | <0.001 | 1972.9 (1355.65, 2871.19) | <0.001 | 48 (1.37) | 123.85 (98.18, 156.23) | <0.001 | 119.62 (95.02, 150.58) | <0.001 | 17 (0.08) | 4.88 (3.16, 7.54) | <0.001 | 4.84 (3.13, 7.47) | <0.001 |
| 2 or 3 | 465 (0.09) | 32 (18.82) | 636.57 (415.69, 974.82) | <0.001 | 626.04 (409.14, 957.93) | <0.001 | 129 (3.68) | 130.52 (112.82, 150.99) | <0.001 | 128.71 (111.28, 148.88) | <0.001 | 91 (0.42) | 8.86 (7.46, 10.53) | <0.001 | 8.74 (7.35, 10.38) | <0.001 |
| 4 to 6 | 4323 (0.80) | 27 (15.88) | 57.81 (36.51, 91.53) | <0.001 | 56.79 (35.78, 90.12) | <0.001 | 804 (22.91) | 84.34 (77.9, 91.31) | <0.001 | 82.91 (76.56, 89.8) | <0.001 | 996 (4.65) | 8.44 (8, 8.92) | <0.001 | 8.31 (7.87, 8.78) | <0.001 |
| 7 or 8 | 23033 (4.28) | 12 (7.06) | 4.82 (2.58, 9.01) | <0.001 | 4.77 (2.56, 8.9) | <0.001 | 1107 (31.55) | 19.33 (17.89, 20.88) | <0.001 | 19.11 (17.68, 20.65) | <0.001 | 3187 (14.87) | 4.31 (4.16, 4.46) | <0.001 | 4.25 (4.10, 4.4) | <0.001 |
| 9 or 10 | 509739 (94.79) | 55 (32.35) | Referent |  | Referent |  | 1421 (40.50) | Referent |  | Referent |  | 17145 (79.98) | Referent |  | Referent |  |
| **Late Term (41^+0^ – 41^+6^wks)** | **N=128336** | **N=51** | **Univariable RRR (95% CI)** | **p-value** | **Adjusted RRR^a^ (95% CI)** | **p-value** | **N=1389** | **Univariable RRR (95% CI)** | **p-value** | **Adjusted RRR^a^ (95% CI)** | **p-value** | **N=7619** | **Univariable RRR (95% CI)** | **p-value** | **Adjusted RRR^a^ (95% CI)** | **p-value** |
| **5 minute Apgar** |  |  |  |  |  |  |  |  |  |  |  |  |  |  |  |  |
| 0 or 1 | 75 (0.06) | 18 (35.29) | 1907.39 (999, 3641.77) | <0.001 | 1771.84 (912.22, 3441.54) | <0.001 | 25 (1.80) | 108.82 (82.18, 144.09) | <0.001 | 105.06 (79.35, 139.09) | <0.001 | 10 (0.13) | 6.32 (3.78, 10.58) | <0.001 | 6.02 (3.65, 9.94) | <0.001 |
| 2 or 3 | 199 (0.16) | 4 (7.84) | 159.75 (53.49, 477.1) | <0.001 | 156.14 (52.44, 464.91) | <0.001 | 74 (5.33) | 100.26 (83.52, 120.35) | <0.001 | 99.59 (82.87, 119.68) | <0.001 | 44 (0.58) | 7.36 (5.81, 9.33) | <0.001 | 7.36 (5.79, 9.35) | <0.001 |
| 4 to 6 | 1549 (1.21) | 10 (19.61) | 51.31 (23.09, 114.02) | <0.001 | 50.54 (22.71, 112.46) | <0.001 | 325 (23.40) | 58.58 (51.76, 66.3) | <0.001 | 57.63 (50.86, 65.3) | <0.001 | 404 (5.30) | 6.74 (6.2, 7.32) | <0.001 | 6.61 (6.07, 7.19) | <0.001 |
| 7 or 8 | 7301 (5.69) | 4 (7.84) | 4.35 (1.45, 13.12) | 0.009 | 4.34 (1.45, 13.02) | 0.01 | 411 (29.59) | 14.02 (12.37, 15.88) | <0.001 | 13.95 (12.31, 15.81) | <0.001 | 1299 (17.05) | 3.82 (3.61, 4.03) | <0.001 | 3.79 (3.58, 4) | <0.001 |
| 9 or 10 | 119212 (92.89) | 15 (29.41) | Referent |  | Referent |  | 554 (39.88) | Referent |  | Referent |  | 5862 (76.94) | Referent |  | Referent |  |
| **Post Term (>42wks)** | **N=7253** | **N=8** | **Univariable RRR (95% CI)** | **p-value** | **Adjusted RRR^a^ (95% CI)** | **p-value** | **N=75** | **Univariable RRR (95% CI)** | **p-value** | **Adjusted RRR^a^ (95% CI)** | **p-value** | **N=444** | **Univariable RRR (95% CI)** | **p-value** | **Adjusted RRR^a^ (95% CI)** | **p-value** |
| **5 minute Apgar** |  |  |  |  |  |  |  |  |  |  |  |  |  |  |  |  |
| 0 or 1 | 10 (0.14) | 3 (37.50) | 1977.37 (224.08, 17448.89) | <0.001 | # |  | 2 (2.67) | 66.2 (19.39, 226.06) | <0.001 | 68.16 (19.93, 233.04) | <0.001 | 0 (0.00) | # | # | # | # |
| 2 or 3 | 12 (0.17) | 1 (12.50) | 551.88 (36.59, 8323.73) | <0.001 | # |  | 6 (8.00) | 139.97 (74.49, 262.99) | <0.001 | 144.1 (76.51, 271.43) | <0.001 | 1 (0.23) | 4.08 (0.71, 23.65) | 0.116 | 4.24 (0.73, 24.55) | 0.11 |
| 4 to 6 | 99 (1.36) | 1 (12.50) | 66.86 (4.21, 1061.6) | 0.003 | ǂ |  | 16 (21.33) | 47.84 (26.87, 85.16) | <0.001 | 46.39 (25.85, 83.26) | <0.001 | 20 (4.50) | 4.98 (3.35, 7.39) | <0.001 | 4.78 (3.19, 7.15) | <0.001 |
| 7 or 8 | 512 (7.06) | 2 (25.00) | 25.86 (2.35, 284.72) | 0.008 | ǂ |  | 24 (32.00) | 13.65 (7.95, 23.44) | <0.001 | 13.54 (7.88, 23.24) | <0.001 | 100 (22.52) | 4.2 (3.42, 5.15) | <0.001 | 4.18 (3.40, 5.12) | <0.001 |
| 9 or 10 | 6620 (91.27) | 1 (12.50) | Referent |  | Referent |  | 27 (36.00) | Referent |  | Referent |  | 323 (72.75) | Referent |  | Referent |  |

N total number (column %); RRR= Relative Risk Ratio; ^a^ Adjusted for pre-eclampsia and body mass index; ^#^ no observations ǂ Model would not converge due to absence of observations in more than 1 subgroup.
