# Supplementary material for: Five-minute Apgar score and risk of neonatal mortality, severe neurological morbidity and severe non-neurological morbidity in term infants – an Australian population-based cohort study
Source: Lancet Reg Health West Pac. 2024 Jan 13;44:101011. doi: 10.1016/j.lanwpc.2024.101011 (PMC10825608; doi:10.1016/j.lanwpc.2024.101011)
Supplement: Appendix [file mmc2.docx]

**Appendix Table 1.** ICD-10 codes relevant to maternal characteristics and neonatal outcomes.

| **Maternal Health and Obstetric History** | **ICD-10 Codes** |
| --- | --- |
| Diabetes Mellitus | E1011, E1015, E1021, E1022, E1023, E1029, E1031, E1033, E1034, E1036, E1040, E1041, E1042, E1043, E1049, E1051, E1061, E1064, E1065, E1069, E1071, E1073, E108, E109, E1101, E1111, E1121, E1122, E1131, E1134, E1136, E1140, E1142, E1143, E1153, E1161, E1162, E1164, E1165, E1171, E1172, E1173, E118, E119, E1322, E1329, E1331, E1342, E1364, E1365, E1371, E1372, E138, E139, E1439, E1443, E1464, E149, G590, O240, O241, O2411, O2412, O2413, O2414, O2419, O242, O2422, O2423, O2424, O2429, O243, O2431, O2432, O2433, O2434, O2439, O244, O2441, O2442, O2443, O2444, O2449, O2453, O2459, O249, O2492, O2493, O2499, O2508, O2509, P700, P701 |
| Preeclampsia | O120, O121, O122, O13, O140, O141, O142, O149, O150, O151, O159, O16, P000 |
| **Neonatal Outcomes** | **ICD-10 Codes** |
| Birth Asphyxia | P200, P201, P209, P210, P211, P219, P110, P111, P112, T71, P916, P211, P9160, P9161, P9163, P9162, R090, P90, P910, P9181 |
| Neonatal Encephalopathy | P916, P9160, P9163, P9162, P9161, P910, P9181 |
| Neonatal Seizures | P90 |
| Intraventricular Hemorrhage | P520, P521, P522, P523, P524, P525, P528, P529 |
| Acidosis at birth (Cord Artery pH <7.0) | E872, P740, P740 |
| Neonatal sepsis | A020, A045, A048, A080, A082, A084, A09, A099, A288, A370, A379, A390, A401, A402, A403, A411, A412, A413, A4152, A4158, A419, A490, A4900, A4901, A491, A492, A493, A498, A499, A500, A502, A509, A851, A86, A870, A878, A879, B004, B005, B007, B009, B019, B259, B332, B341, B343, B349, B369, B370, B372, B377, B379, B389, B390, B432, B49, B951, B952, B954, B9542, B9548, B955, B956, B957, B958, B961, B962, B9639, B965, B966, B967, B968, B970, B971, B973, B974, B976, G001, G002, G008, G009, G039, G049, G051, G060, G08, I38, I400, I514, J111, J121, J13, J151, J180, J189, J210, J219, J22, J869, K612, K631, K750, L028, L0302, L0311, L033, L038, L039, L089, M0096, P230, P231, P232, P233, P234, P235, P236, P238, P239, P240, P249, P350, P351, P352, P353, P358,  P359, P360, P361, P362, P363, P364, P365, P368, P369, P371, P372, P373, P375, P3750, P3751, P3752, P3759, P378, P379, P38, P390, P391, P392, P393, P394, P398, P399, P77, P780, P781, R572, T814, Z0371 |
| Birth Trauma | P100, P101, P104, P108, P111, P112, P113, P120, P121, P122, P123, P124, P128, P129, P131, P133, P134, P140, P143, P152, P153, P154, P158, P159, P110, P115, P130, P132, P138, P139, P141, P150, P155, P103, P119, P102,  P109, P156, P148, S020, S021, S022, P142, P151, S059, S065 |
| Necrotizing Enterocolitis | P77, P780 |
| Hypoglycemia | P703, P704 |
| Hypothermia | P800, P808, P809, P810, P818, P819 |
